# Tracheal Length Measurement in Intubated Neonates to Guide the Design and Use of Endotracheal Tube Glottic Depth Markings

**DOI:** 10.3390/children9020169

**Published:** 2022-01-29

**Authors:** Jennifer B. Cerone, Joaquim M. B. Pinheiro

**Affiliations:** Department of Pediatrics, Albany Medical Center, Albany, NY 12208, USA; ceronej@amc.edu

**Keywords:** neonates, trachea, growth and development, tracheal tubes, intubation, measurements, safety, design standards

## Abstract

Background: Data on neonatal tracheal length are needed to inform the standardization of safety features for endotracheal tubes (ETTs) such as glottic depth markings. Laryngotracheal airway measurements are available from digital imaging in infants and children but not in neonates. We aimed to determine the tracheal length (TL) of intubated preterm and term neonates. Methods: An observational study was performed on 57 neonates of 22–42 weeks’ gestation and <1 week of age. Two clinicians independently reviewed 153 digital chest radiographs to determine the carina position and TL. TL was measured from carina to mid-C4 (cricoid level). We analyzed interrater agreement (within 0.5 vertebral levels) on the position of the carina and TL. TL was plotted as a function of gestational age and weight, using graphical and regression analyses. Results: Carina position ranged from T3 to T5.5, with an interrater agreement of 95%. On image pairs concordant for carina position, TL determinations were virtually identical between readers (mean difference 0.1 mm, 95% CI −0.5–0.6 mm). Average mid-tracheal length overlies the body of T1. In infants aged less than 32 weeks’ gestation, the mid-trachea lies <20 mm from the carina or the larynx. TL linearly correlates with gestational age, but correlation with birthweight best fits a segmented regression with a node at 1 kg. Conclusions: The functional length of the laryngotracheal airway can be reliably measured in sick neonates. It correlates well with gestational age and birthweight, and this information can inform the redesign of ETT markings to promote the safer use of these devices.

## 1. Introduction

Endotracheal tubes (ETTs) are commonly used in critically ill neonates, and their optimal placement is essential to provide adequate ventilation and minimize injury or harm to the infant. The short length of the neonatal trachea allows for very narrow tolerances in the safe position of endotracheal tubes [1]. A shallow position with consequent unplanned extubation and deep position resulting in endobronchial intubation are both frequent safety events associated with substantial risks in clinically fragile newborns [2,3,4]. Maturational growth, biologic variation, cyclic changes during normal respiration, ventilatory assistance, head flexion or rotation, and movement of the ETT holding device all affect the functional length of the airway and position of the ETT tip [5,6,7].

Determining the intended ETT depth prior to intubation and using the vocal cord guide marking(s) on the ETT during intubation are the two main strategies aimed at accurately positioning the ETT near the mid-trachea [8]. Several different methods are used to predict the depth of ETT insertion including those based on weight or gestational age (GA) and nasal–tragus length [9,10]. Published recommendations for appropriate ETT depth based on the neonate’s weight or gestational age vary significantly [11,12,13,14], and the original formula proposed by Tochen et al. [15] is inaccurate in extremely preterm neonates [11,12,16].

Given the varying and unsatisfactory guidelines for predicting ETT depth, markings to guide ETT insertion to the vocal cord level under direct visualization have also been used to aid in safe tube positioning. In 1974, Loew et al. [17] studied the use of a single black line guiding ETT insertion to the level of the vocal cords located at 2.2, 2.4, and 2.6 cm from the ETT tip for nominal size tubes 2.5, 3.0, and 3.5, respectively. Vocal cord markings vary among ETT manufacturers [18,19,20,21] and package inserts have provided no guidance to the caregivers on the proper use of such markings; both of these factors contribute to user uncertainty and error in accurate ETT placement [8]. Improving safety standards for ETT design should be based on neonatal airway dimensions [17,18,19,22,23] but such measurements have been scarce and discrepant between studies, particularly those comparing living preterm neonates and deceased fetuses [5,23,24,25,26]; there is evidence that postmortem tracheal length is significantly shorter than that in the living, dynamic state [27]. Due to insufficient evidence on the relative efficacy of various ETT markings from different manufacturers, the International Organization for Standardization (ISO) has not standardized these markings [28].

The availability of digital imaging has allowed in vivo measurements of the laryngotracheal airway in infants and young children, which could inform ETT design. However, no such data are available for living neonates [29]. Thus, the purpose of this study was to determine the tracheal length (TL) of preterm and term neonates in vivo from digital radiographs, while testing the hypothesis that TL is predictable from weight or GA.

## 2. Materials and Methods

This was an IRB-approved single center observational, descriptive study of radiographs obtained from March 2012 through December 2013 conducted in the Neonatal Intensive Care Unit (NICU) at Albany Medical Center, a level IV regional perinatal center. All intubated neonates less than one week of age with digital chest radiographs were eligible, spanning a wide range of gestational ages from 22 to 42 weeks. We excluded any digital radiographs with tension pneumothoraces, marked rotation or other conditions in which there was significant distortion of the airway. Digital chest radiographs were retrospectively and independently reviewed by both authors to determine the position of the carina relative to the vertebrae and to measure tracheal length.

### 2.1. Measurement of Tracheal Length

**Figure 1:**
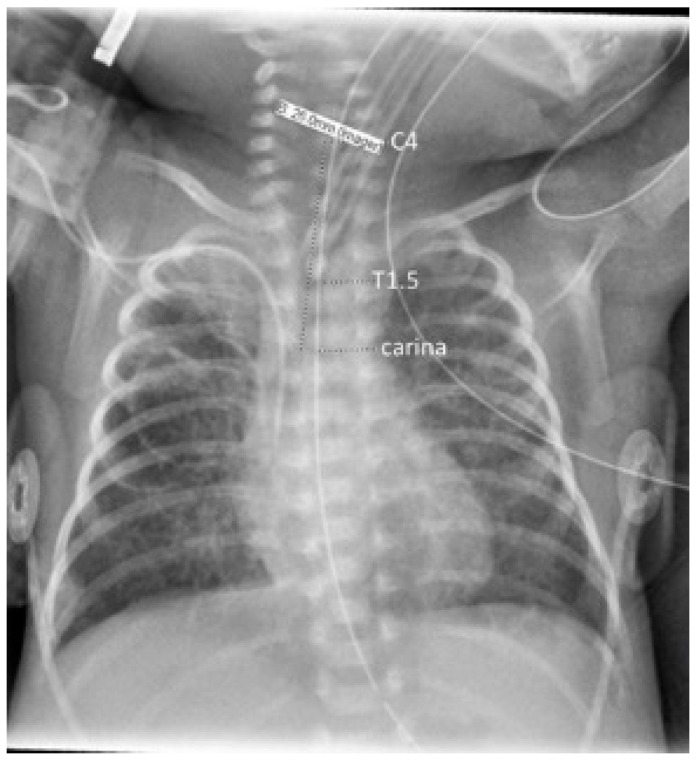
Chest radiograph illustrating annotations of anatomical landmarks with the dashed line markers, underlying the measurements; tracheal length was taken as the carina–C4 distance.

Vertebral bodies were identified by counting ribs from the first to the 12th (if present), thus designating T12, before counting the cephalad to locate the carina, T1, and C4. Carina position was used in reference to the vertebral level on an interval scale, with interspaces designated as half levels. Tracheal length was measured from carina to mid C4, the neonatal cricoid level [30,31], as shown in Figure 1. The cursor built into the imaging software (IntelliSpace PACS Enterprise 4.4, Philips Medical Systems, Amsterdam, The Netherlands) was then used to measure the distances from carina to mid C4 length (tracheal length), carina to mid-T1, and carina to T1.5 (T1–T2 interspace), to the nearest mm. T1 has been shown by Thayyil et al. to be a superior radiological reference marker for the neonatal mid-trachea [32]. T1.5 has been used in our clinical quality improvement work as the operationally defined target for the ETT tip; however, acceptable positions of the tube tip (requiring no adjustment) range between the top of T1 and the bottom of T2. The software’s contrast limited adaptive histogram equalization (CLAHE) tool was applied to images to optimize contrast for the visualization of the carina.

### 2.2. Statistical Analysis

Demographic data collected included gestational age, postnatal age, and weight. Images were temporarily identified by the patient medical record number, date, and time of the study, to allow for independent duplicate readings of the same radiographs on the imaging system; the final dataset for analysis included only a study number, the baby’s gestational and postnatal ages and weight, and the tracheal dimensions obtained independently by each reader. All statistical and graphical analyses were conducted using Stata 14 software (College Station, TX, USA). Linear regression was applied first, then polynomial regression, and ultimately segmented regression was used to describe the weight–tracheal length relationship, as well as the relationship between weight and the C4–T1 length. The regression parameters were used to plot prediction lines for these relationships; distances from the ETT tip to the distal glottic marking, overlaid on these plots, were based Loew et al. [17] for 2.5–3.5 ETT sizes, and on the package insert specifications for the 2.0 ETT size (Shiley™ 2.0 mm ID, Covidien LLC, Mansfield, MA, USA).

## 3. Results

**Figure 2:**
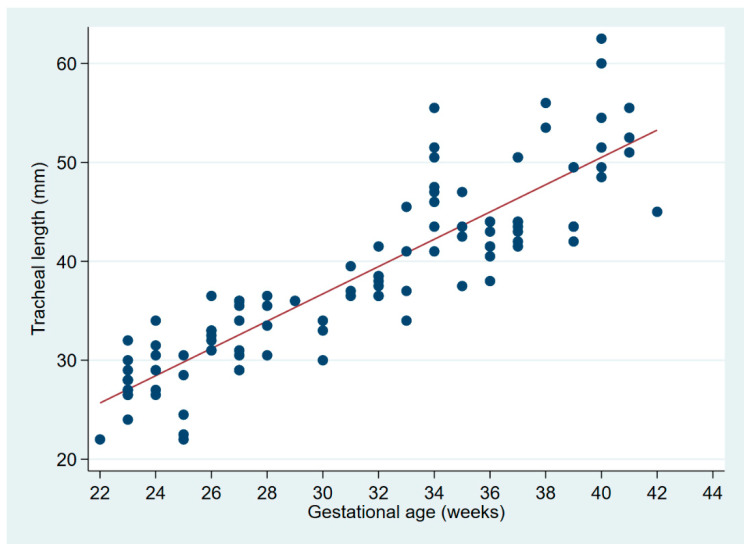
Linear regression of tracheal length on gestational age. The regression equation is: tracheal length (mm) = −1.9 + 1.3 × GA (weeks), with r² = 0.7234 and *p* < 0.0001.

The two authors independently reviewed 153 digital chest radiographs from neonates at 22–42 weeks’ gestation, among which 114 were in the first week. Six radiographs were excluded from analysis because the carina could not be visualized; or, ribs and vertebral bodies could not be counted consistently due to exposure or overlying equipment, leaving 108 radiographs for the present tracheal length analysis. The mean birth weight was 1895 g (range, 440–4500 g), and GA 31.2 weeks (range 22–42 weeks). Carina positions ranged from T3 to T5.5 (Supplementary Figure S1, histogram), with a median at T4 (IQR T4–T4.5). The two readers agreed on the position of the carina in 139 (95%) of the 147 radiographs in which the carina was identifiable. On image pairs concordant for carina position, TL determinations were virtually identical between readers (mean difference 0.1 mm, 95% CI −0.5–0.6 mm). TL ranged from 22 to 64 mm. There is a linear relationship between TL and gestational age (Figure 2).

**Figure 3:**
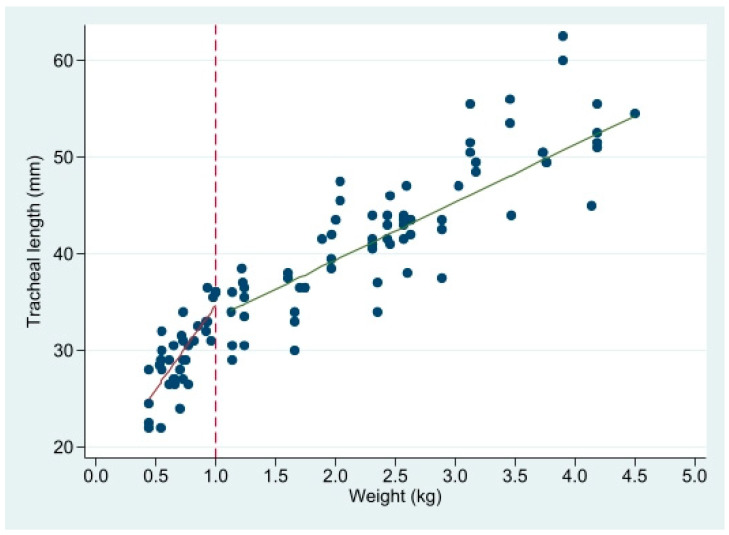
Linear regression of tracheal length (mm) on weight, in 2 segments with a node at 1 kg. See Table 1 for regression parameters.

TL related to weight is better fitted by a segmented regression with a node at weight = 1 kg (Figure 3 and Table 1).

The average mid-tracheal length, calculated as half of the C4–carina tracheal length, overlies the body of T1, although in smaller neonates, the carina–T1 distance tends to be shorter than the mid-tracheal distance (Supplementary Figure S2A). The carina–T1.5 distance is consistently shorter than the mid-tracheal distance (Supplementary Figure S2B), i.e., T1.5 is usually caudad of the mid-trachea.

Plotting the C4–T1 length, as well as the tracheal length as a function of weight, and comparing these with the expected position of the ETT tip if standard distal glottic depth marks are used (21–26 mm for ETT sizes 2.0 to 3.5, respectively), the ETT tip is expected to rest beyond T1 in most neonates weighing <2 kg, and near the carina (full tracheal length) in the smallest viable neonates. Of note, as extrapolated from Figure 2, the mid-trachea in infants less than 32 weeks’ gestation lies <20 mm from the carina or the larynx.

## 4. Discussion

The optimal positioning of ETT is imperative for the safer care of critically ill neonates, especially those prematurely born and at earlier gestational ages. The mid-trachea is the preferred location for ETT placement during NICU care, to minimize endobronchial migration, while also avoiding unplanned extubations. Similarly to other studies, we found that the average mid-tracheal position overlies T1 [33] and the mid-tracheal length lies less than 20 mm from the carina and the larynx in infants less than 32 weeks’ gestation. Given this narrow tolerance for ETT tip movement, the use of a distal vocal cord mark to guide ETT depth as studied by Loew et al. [17] at 22, 24 and 26 mm could contribute to endobronchial intubation in smaller preterm neonates. Balu et al. [34] described their experience with vocal cord markings spanning from 7 to 25 mm on ETTs irrespective of tube size leading to inadvertent deep intubation in extremely premature infants. In the study of Balu et al., the authors measured C4–T2 length on X-rays to estimate the mid-tracheal distance, thus adding 1 vertebral level to our own measurements. One vertebral level (intervertebral disc plus vertebral height) measures between 5 and 8 mm from the smallest to the largest neonates (J. Pinheiro, unpublished). Adjusting for this difference, and visually comparing our data on mid-tracheal length as a function of weight with those of Balu et al., we note a remarkable similarity in the distribution of data points, with our mid-tracheal lengths appearing to be approximately 3–5 mm shorter than theirs, ranging from smaller neonates (440 g and 22 weeks’ gestation, in our study) to larger ones.

**Figure 4:**
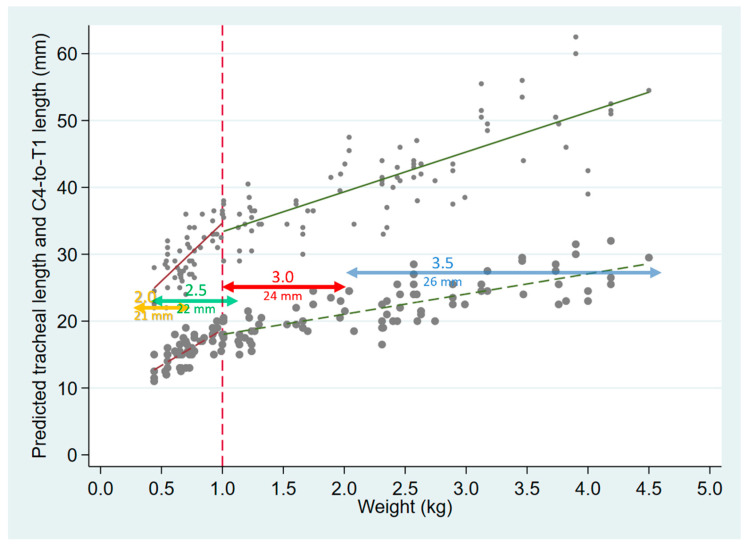
Tracheal length (small gray circles, with solid regression lines as in Figure 3), and C4–T1 length (larger gray circles and corresponding dashed regression lines), both plotted as a function of weight. The horizontal, colored lines are plotted at distances corresponding to the tip-distal glottic depth mark on ETTs sizes 2.0–3.5, with the horizontal span of the lines representing the approximate range of infant weights for whom each ETT size is appropriate.

A recently published randomized study showed no difference between the use of vocal cord guidelines versus weight-based estimations (weight in kg + 6 cm) in the accurate positioning of ETTs [35]. In that study, an ETT model with a single glottic depth mark was used; however, 36% of ETTs were positioned too deep, beyond T2. Endotracheal tubes from different manufacturers have varying types of markings consisting of single and multiple lines located at discrepant distances from the ETT tip, increasing confusion among users [19,23]. A survey of neonatal care practitioners, chiefly comprising experienced neonatologists, showed that they were uncertain regarding the proper use of vocal cord reference markings on ETTs [8]. Using the double-line marking which is 1 cm proximal to the single distal line (thus located at 32 and 36 mm from the tip in ETT sizes 2.5 and 3.5, respectively) further increases the likelihood of endobronchial positioning. Using a size 2.0 ETT in which the single distal line is at 21 mm from the tip, and the double line at 33 mm, insertion to the double line at the vocal cords of neonates in the 300–600 g weight range would result in tip placement well past the mid-trachea, and often at or beyond the carina, as apparent in Figure 4. The current Neonatal Resuscitation Program (NRP) Textbook recommendations are unclear regarding which glottic marks should be used [14], but an online training video clearly suggests inserting the ETT past the single line, to the double line at the cords (https://www.youtube.com/watch?v=v92u23ZZOho&feature=youtu.be, at 04:26 on the video; accessed 27 December 2021) However, when unplanned extubation is especially undesirable, as in some situations under anesthesia, positioning the ETT towards the lower end of the trachea may be preferred [28].

Several investigators have sought to determine accurate estimations of ETT insertion depths based on crown–rump length, crown heel length, foot length, gestational age, and weight [6,36,37]. Hipolito et al. [38] used weight to derive expected ETT insertion depth, and proposed four brightly colored markings at 6.5–9.5 cm from the ETT tip, to guide positioning at the mid upper gingival line. Recently, postmortem magnetic resonance imaging has been used to measure airway dimensions; such data could be applied to a web-based application using gestational age and weight to predict mid-tracheal length and inform ETT insertion depth [36]. Others have also proposed a formula combining birthweight and gestational age to determine the ideal depth of insertion for ETT in neonates [16].

Similarly to our findings, Kempley et al. [39,40] showed a linear relationship between gestational age and tracheal length to ensure adequate ETT depth. These authors caution against the use of Tochen’s (7–8–9) rule [15] or any derivative of a weight-based formula to estimate ETT depth and recommend the use of a gestational age-based table to guide intubation practice. Based on our findings, although birthweight also correlates with tracheal length, this relationship is segmented at 1 kg, leaving extremely premature infants at risk of malpositioned ETTs when using linear weight-based calculations to determine insertion depth. Bartle et al. [41] also found their institutional weight based formula to be inaccurate for infants weighing less than 1 kg albeit their formula was an improvement over the former weight-based NRP guidelines. Another recent study derived a weight-based formula (3.5 × birthweight (kg) + 3.6 cm) for infants weighing less than 750 g; their formula yields estimated depths of insertion that are similar to the current NRP recommendations [14], but they found no correlation with gestational age [42].

The findings of our study are concordant with those of Rigo et al. [23], whose postmortem measurements from 114 fetuses and neonates were used to derive calculations of vocal cord to mid-tracheal length based on gestational age, weight, and length—concluding that gestational age had the best correlation. They also proposed that ETT depth markings should be colored, based on gestational age and weight to ensure optimal positioning. Their very precise recommendations for ETT depth markings result in marking positions that start at 17.7 mm, and do not reach 22 mm until 32 weeks’ gestation; these data are consistent with our findings, suggesting that present ETT glottic marks promote an overly deep positioning of the ETT tip in very preterm neonates. Our study adds such measurements in vivo, from standard digital chest radiographs, further supporting the need for revised and standardized ETT vocal cord markings.

Our study has several limitations. We did not collect data on body length as a potential correlate of tracheal length, since most studies use gestational age and weight, and our NICU’s emergency drug doses and procedural guidelines are based on weight; furthermore, when the study was planned, our NICU dietitians had concerns that body length measurements were unreliable in our setting [43]. Another potential shortcoming is that we do not routinely adjust head position for radiographs, for multiple reasons related to patient safety; instead, we excluded radiographs with significantly distorted images; in any case, head flexion and rotation is known to affect distal ETT position within, but not necessarily tracheal length.

A particularly important limitation of our study and others relying on radiographs is that C4–carina length provides only an indirect estimate of tracheal length, and it does not necessarily equal vocal cord–carina length. The use of the C4 vertebra as a marker for the cricoid or lower laryngeal level in neonates is primarily derived from Noback’s study [30], and supported by scant subsequent evidence [31], but we found no disconfirming data that challenge this assumption. If we further wish to account for distance to the vocal cords, we can rely on the autopsy data from Fayoux et al. [5], and add between 4 and 7 mm (depending on the neonate’s size) to the C4–carina measurement. Because of the uncertainties inherent in estimating the vocal cord–carina distance from the present data, we did not attempt to derive a precise recommendation for the redesign of glottic depth marks on ETTs. Nevertheless, our results, in agreement with Balu et al. [34] and further supported by Rigo et al. [23], suggest that inserting the commonly used single-line glottic depth mark to the vocal cord level will place the ETT tip deeper than the mid-trachea, in extremely preterm neonates, and this has significant implications for neonatal intubation practice guidelines.

## 5. Conclusions

The functional length of the laryngotracheal airway can be reliably measured in sick neonates using readily available digital radiographs, and TL correlates well with both gestational age and birthweight, albeit with a strikingly different linear relationship in infants weighing less than 1 kg. This supports the current NRP guidelines which provide tables with ETT insertion depth recommendations based on gestational age and non-linear weight categories [14]; however, our data indicate that the distal glottic mark (single), and not the double line, should be routinely used to guide ETT insertion depth at the vocal cords. Furthermore, the short tracheal lengths should guide intubation practice, particularly the redesign and use of glottic depth markings on currently available ETTs. As a first step, the recent update of the ISO standard on tracheal tubes requires that individual ETT packages display the distance between the glottic depth marks and the tube tip [28]. This should immediately increase awareness of the markings by clinicians at all levels of expertise and thus promote safer ETT use. Further updates can use data from recent studies including ours to refine ETT design standards, improve the usability of these devices, and thus promote safer neonatal intubation practices.

## Figures and Tables

**Table 1:** Segmented linear regression of tracheal length (mm) on weight, with a node at 1 kg ^1^.

Factor	Coefficient	Std Err	*p*	(95% CI)
Weight < 1 kg	17.3	2.9	<0.001	(11.6, 23.2)
Weight > 1 kg	6.0	0.4	<0.001	(5.2, 6.8)
Intercept2	−1.3	1.2	0.267	(−3.6, 1.0)
Constant	17.3	2.2	<0.001	(12.9, 21.7)

^1^ Regression r^2^ = 0.8026; *p* < 0.0001.

## Data Availability

Deidentified data are available upon written request to the corresponding author.

## Supplementary Materials

The following are available online at https://www.mdpi.com/article/10.3390/children9020169/s1, Figure S1: Frequency distribution of the carina level; Figure S2: Carina–mid-tracheal length.
