# Supplementary material for: Tracheal Length Measurement in Intubated Neonates to Guide the Design and Use of Endotracheal Tube Glottic Depth Markings
Source: Children (Basel). 2022 Jan 29;9(2):169. doi: 10.3390/children9020169 (PMC8870647; doi:10.3390/children9020169)

DOI: 10.5281/zenodo.5879916
Cerone, Jennifer B., & Pinheiro, Joaquim M. B. (2021). Tracheal length in intubated neonates - supplemental figures. Zenodo. https://doi.org/10.5281/zenodo.5879916
HTTPS://ZENODO.ORG/RECORD/5879916#.YCOLO2JMLRY

SUPPLEMENTAL FIGURE S1:
Histogram showing the frequency distribution of the carina level, ranging from T3 to T5.5

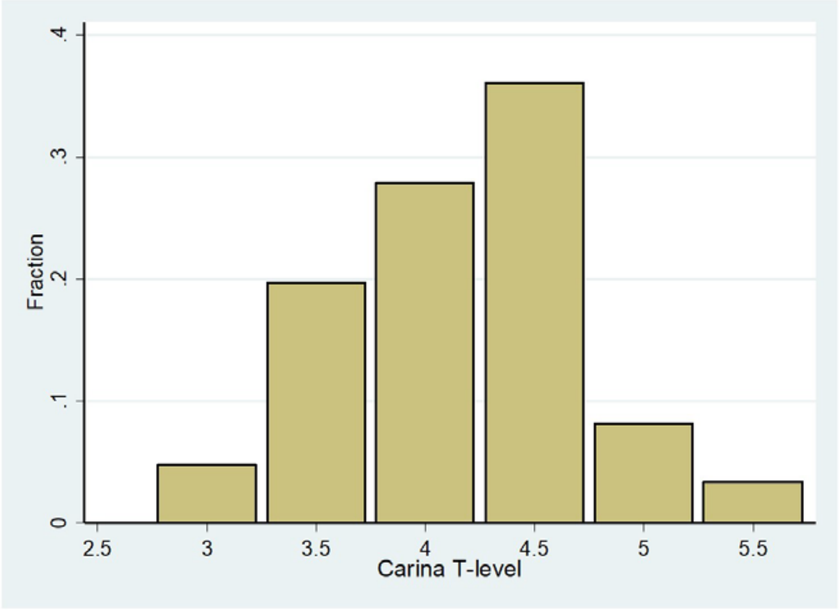

SUPPLEMENTAL FIGURE S2:
Carina to mid-tracheal length, assuming that the mid-trachea overlies either the T1 or T1.5 vertebral levels. Dashed line is identity, where mid-tracheal length would equal carina to mid-T1 length, or carina to T1.5 length.

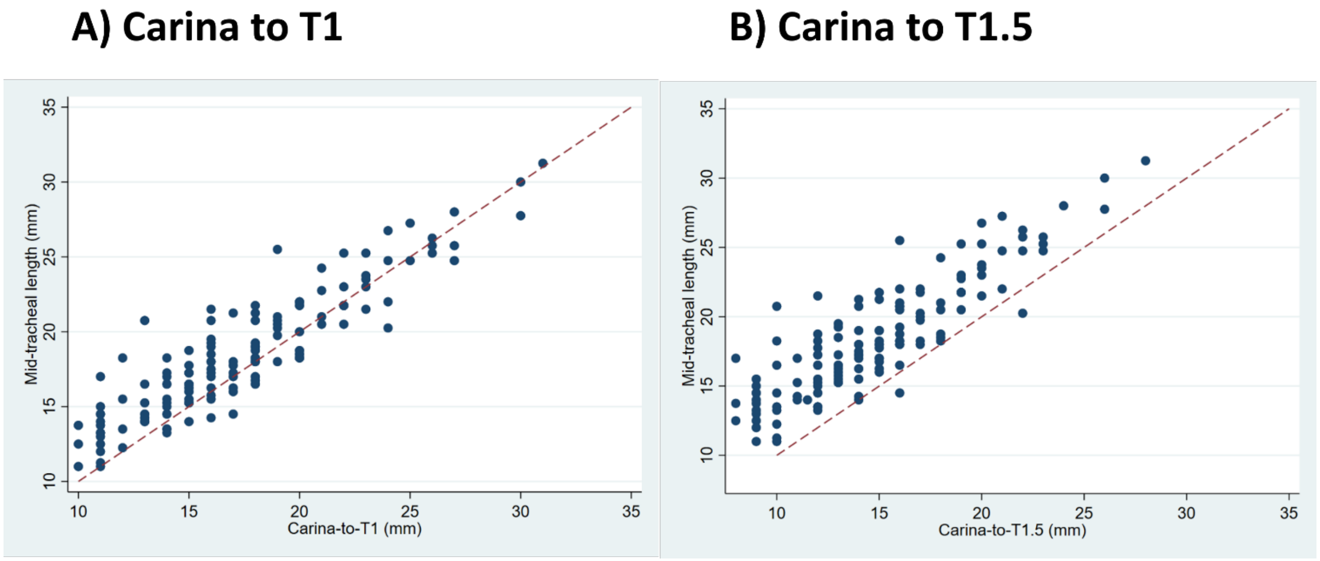

Supplement: Supplementary file 1 [file children-09-00169-s001.zip › children-1545297-supplementary.pdf]
